# Ranging patterns, spatial overlap, and association with dolphin morbillivirus exposure in common bottlenose dolphins *(Tursiops truncatus)* along the Georgia, USA coast

**DOI:** 10.1002/ece3.4727

**Published:** 2018-11-26

**Authors:** Brian Balmer, Eric Zolman, Teri Rowles, Cynthia Smith, Forrest Townsend, Deborah Fauquier, Clay George, Tracey Goldstein, Larry Hansen, Brian Quigley, Wayne McFee, Jeanine Morey, Patricia Rosel, Jerry Saliki, Todd Speakman, Lori Schwacke

**Affiliations:** ^1^ Jardon and Howard Technologies (JHT) Incorporated Orlando Florida; ^2^ National Oceanic and Atmospheric Administration, National Ocean Service National Centers for Coastal Ocean Science Charleston South Carolina; ^3^ National Marine Mammal Foundation San Diego California; ^4^ National Oceanic and Atmospheric Administration, National Marine Fisheries Service Office of Protected Resources Silver Spring Maryland; ^5^ Bayside Hospital for Animals Fort Walton Beach Florida; ^6^ Georgia Department of Natural Resources Nongame Wildlife Conservation Brunswick Georgia; ^7^ Karen C. Drayer Wildlife Health Center, One Health Institute, School of Veterinary Medicine University of California Davis California; ^8^ National Oceanic and Atmospheric Administration, National Marine Fisheries Service Southeast Fisheries Science Center Lafayette Louisiana; ^9^ Athens Veterinary Diagnostic Laboratory University of Georgia Athens Georgia

**Keywords:** bottlenose dolphin, morbillivirus, movement patterns, spatial overlap, telemetry

## Abstract

During 2013–2015, an outbreak of dolphin morbillivirus (DMV) occurred in the western North Atlantic, which resulted in the stranding of over 1,600 common bottlenose dolphins *(Tursiops truncatus)*. There are currently five coastal and 10 bay, sound, and estuary dolphin stocks along the U.S. Atlantic coast, yet there is very limited understanding of which stocks were exposed to DMV during the recent outbreak, or how DMV was transmitted across stocks. In order to address these questions, information is needed on spatial overlap and stock interactions. The goals of this project were to determine ranging patterns, prevalence of DMV, and spatial overlap of the South Carolina‐Georgia (SC‐GA) Coastal Stock, and adjacent Southern Georgia Estuarine System (SGES) Stock. During September 2015, a health assessment and telemetry study was conducted in which 19 dolphins were captured, tested for antibodies to DMV, and satellite tagged. Dolphins were classified into one of three ranging patterns (Coastal, Sound, or Estuary) based upon telemetry data. Coastal dolphins (likely members of the SC‐GA Coastal Stock) had a significantly higher prevalence of positive DMV antibody titers (0.67; *N* = 2/3), than Sound and Estuary dolphins (likely members of the SGES Stock) (0.13; *N* = 2/16). These results suggest that the SC‐GA Coastal Stock may have experienced greater exposure to DMV as compared to the SGES Stock. However, due to the small size of the SGES Stock and its exposure to high levels of persistent contaminants, this stock may be particularly vulnerable to DMV infection in the future.

## 1 INTRODUCTION

During a 10‐month period between June 1987 and March 1988, an outbreak of cetacean morbillivirus in the western North Atlantic led to the stranding of at least 667 common bottlenose dolphins *(Tursiops truncatus) *along the U.S. Atlantic coast from New Jersey southward to Florida (Geraci, 1989; McLellan, Friedlaender, Mead, Potter, & Pabst, 2002). Recently, another major die‐off occurred along the same span of the U.S. Atlantic coast in which over 1,600 common bottlenose dolphins stranded between July 2013 and March 2015 (NMFS unpub. data, Morris et al., 2015). The apparent cause of the significant increase in stranded common bottlenose dolphins, which represents an 8‐fold increase over historical stranding rates, was determined to be dolphin morbillivirus (DMV) infection (Morris et al., 2015; Van Bressem et al., 2014). Currently, it is unclear why DMV outbreaks periodically impact common bottlenose dolphins in the western North Atlantic. Possible factors may include reintroduction of the virus associated with natural cyclic fluctuations of “herd immunity” (Duignan et al., 1996), changes in disease transmission associated with dolphin migrations, and spatial overlap between populations of small cetaceans. To assess factors associated with DMV transmission among common bottlenose dolphins in the western North Atlantic, a better understanding of movement patterns, spatiotemporal overlap of stocks, and assessment of exposure to DMV within stocks is needed.

**Figure 1:**
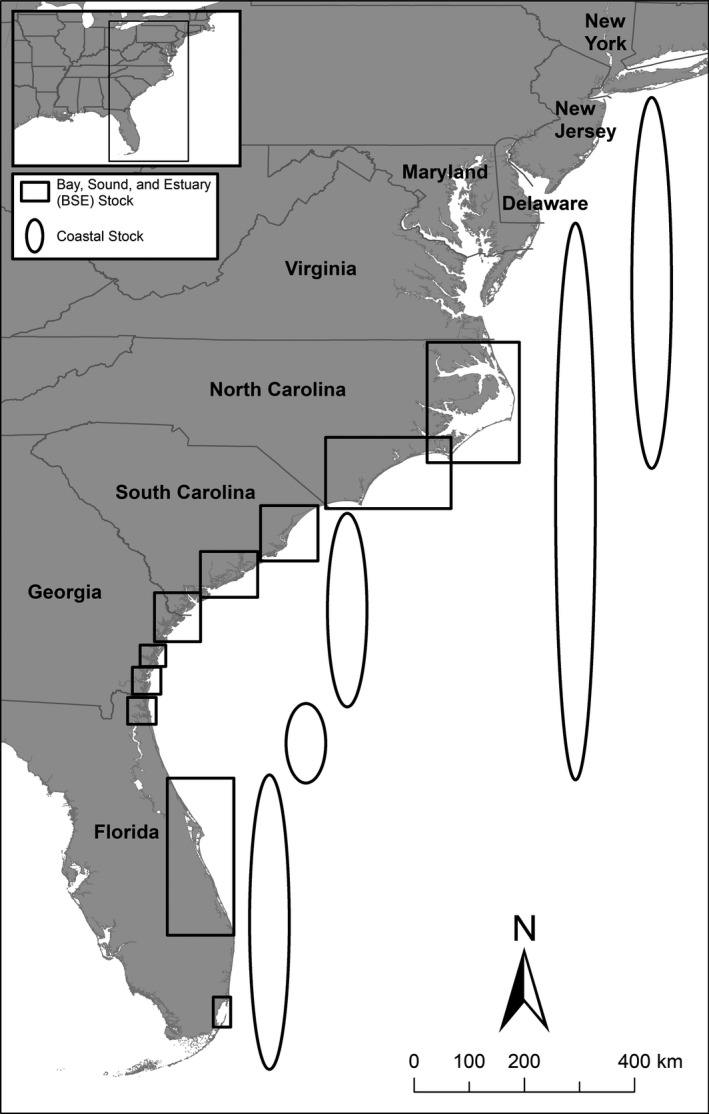
Western North Atlantic common bottlenose dolphin bay, sound, and estuary (BSE), and coastal stock structure (adapted from Hayes et al., 2017). BSE stock boundaries include the estuarine and nearshore waters (≤1 km from shore). Coastal stock boundaries include primarily estuarine and inshore waters (≤20 m deep) with evidence for some coastal stocks to extend into continental shelf waters

The U.S. Marine Mammal Protection Act defines a stock as “…a group of marine mammals of the same species in a common spatial arrangement that interbreed when mature” (Marine Mammal Protection Act 16 U.S.C. 1,361 et seq.). Stock definitions ensure that conservation efforts to mitigate human activity are aimed at the appropriate management unit (reviewed in Conn, Gorgone, Jugovich, Byrd, & Hansen, 2011). Common bottlenose dolphin stock structure in the western North Atlantic is a complex mosaic of overlapping bay, sound, and estuary (BSE), and coastal stocks (Hayes, Josephson, Maze‐Foley, & Rosel, 2017) (Figure 1). From North Carolina to Florida, there are 10 BSE stocks currently recognized (Hayes et al., 2017), characterized by year‐round residency, high site fidelity, and localized ranging patterns (e.g., Zolman, 2002, Read, Urian, Wilson, & Waples, 2003, Mazzoil et al., 2008, Balmer et al., 2013). Concurrently, five coastal stocks (Northern and Southern Migratory, South Carolina‐Georgia, Northern Florida, and Central Florida Coastal Stocks) have been designated (Hayes et al., 2017). The Northern Migratory Coastal Stock extends as far north as New York in the summer and as far south as Cape Hatteras, North Carolina in winter. The Southern Migratory Coastal Stock extends as far north as the eastern shore of Virginia during summer and as far south as northern Florida in winter. The three other coastal stocks (South Carolina‐Georgia, Northern Florida, and Central Florida) include dolphins present in these respective waters but not part of adjacent BSE or the migratory coastal stocks, and are not thought to migrate seasonally. Currently, ranging patterns and site fidelity of the latter three coastal stocks are not well understood. Strandings from the 2013–2015 DMV outbreak were thought to be primarily from the Northern and Southern Migratory Stocks, the South Carolina‐Georgia Coastal Stock, and the Northern Florida Coastal Stock (Morris et al., 2015). Strandings from three BSE stocks, one in Northern North Carolina and two in Florida from Jacksonville and Indian River Lagoon, were also documented to be positive for DMV (NMFS unpublished data), but is not known whether infection occurred in other BSE stocks due to lack of data.

Aerial survey, genetic, small vessel photographic‐identification (photo‐ID), stranding, and telemetry data have provided insight into coastal dolphin ranging patterns and spatial overlap with BSE stocks (reviewed in Hayes et al., 2017). Several sites in South Carolina and Georgia have been relatively well‐studied and found to experience seasonal increases in dolphin abundance during summer and fall suggestive of individuals from coastal stocks entering BSE waters (Balmer et al., 2013; Speakman, Lane, Schwacke, Fair, & Zolman, 2010). During the 1987–1988 and 2013–2015 mortality events, temporal stranding patterns (McLellan et al., 2002) and long‐term photo‐ID data (NMFS unpub. data, Urian, 2016) suggested that the Northern and Southern Migratory Coastal Stocks were the first stocks impacted by both of the DMV outbreaks. As these stocks migrated along the western North Atlantic coast, animals may have spread the disease to other coastal and/or BSE stocks causing the mortality events to extend spatially along the coast over time.

Migratory animals experience high energetic demands that may reduce immune function and increase susceptibility to disease (reviewed in Bowlin et al., 2010). These factors make migrating animals potential sources for infectious diseases that can in turn expand the geographic distribution of a pathogen or mortality event (Altizer, Bartel, & Han, 2011). Diseases, such as DMV, are thought to be transmitted between animals via inhalation or direct contact (Black, 1991; Van Bressem, Waerebeek, & Raga, 1999) and contact rates are generally higher in social species than solitary species (Craft, Hawthorne, Packer, & Dobson, 2008). Positive correlations between animal contact and spatial overlap of hosts have been identified in numerous species across many taxa and may affect disease transmissions among animals (reviewed in Robert, Garant, & Pelletier, 2012). Within a species, disease transmission rates may vary by pathogen (or strain) (Mideo, Alizon, & Day, 2008), age‐class (Greig, Gulland, & Kreuder, 2005), group size (Côté & Poulinb, 1995), migration pattern (Altizer et al., 2011), season (Rogers et al., 1998), or sex (Creel & Creel, 1991).

A model for DMV transmission during the recent 2013–2015 mortality event was recently developed, and model analyses suggest that frequency‐dependent transmission predominantly regulated the outbreak (Morris et al., 2015). Common bottlenose dolphins are highly sociable and have a fission–fusion society in which group composition can change quickly on an hourly to daily basis (reviewed in Connor, Wells, Mann, & Read, 2000). In the western North Atlantic, common bottlenose dolphin group size and cohesion tend to be greater in coastal as opposed to BSE environments (Speakman et al., 2006; Toth, Hohn, Able, & Gorgone, 2011), which may suggest different contact rates and potential differences in disease transmission rates within and across stocks. However, little is known about degree of interaction and possible spatiotemporal overlap between coastal and BSE stocks, both of which are essential for assessing contact rates and disease transmission.

A confluence of common bottlenose dolphin stocks occurs along the Georgia coast, where the ranges of five stocks, the Southern Migratory Coastal Stock, the South Carolina‐Georgia (SC‐GA) Coastal Stock, and adjacent Northern, Central, and Southern Georgia Estuarine System Stocks, overlap. The goals of the current study were to examine ranging patterns and spatial overlap of two of these stocks, the South Carolina‐Georgia (SC‐GA) Coastal Stock, and adjacent Southern Georgia Estuarine System (SGES) Stock, and to assess DMV antibody prevalence and pathogen presence through capture‐release sampling and satellite telemetry.

## 2 MATERIALS AND METHODS

### 2.1 Study site

**Figure 2:**
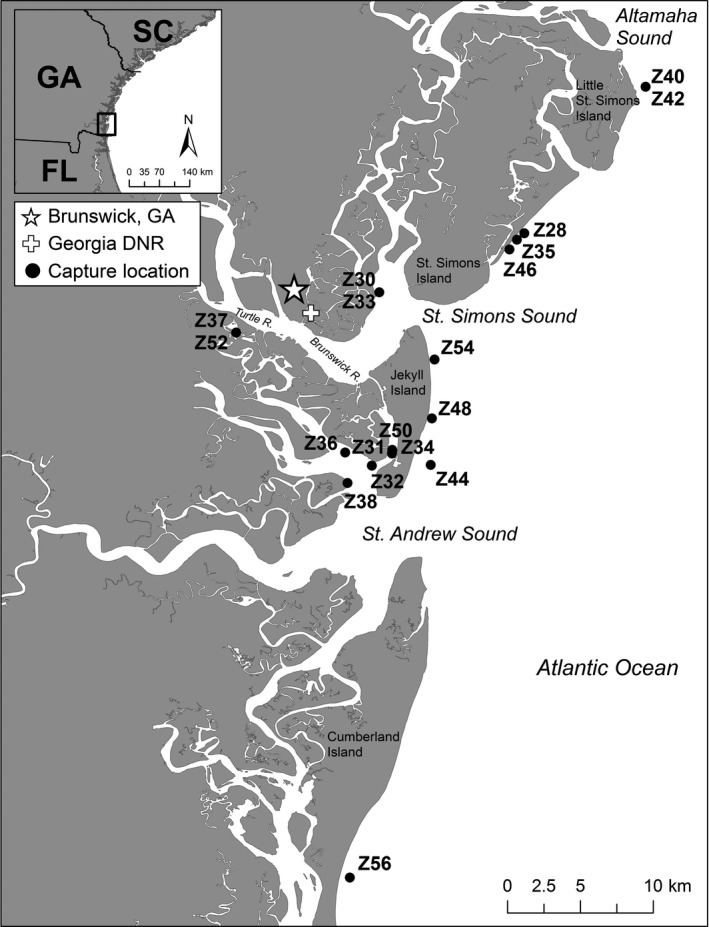
Georgia health assessment and telemetry study area and capture locations for free‐ranging common bottlenose dolphins (*N* = 19). Letter (Z) and two‐digit number (♀, odd; ♂, even) are identifiers for individual tagged dolphins

The boundaries for the SGES and SC‐GA Coastal Stocks are the BSE waters from the Georgia‐Florida state line (St. Marys Entrance) northward to Altamaha Sound, and all of the South Carolina and Georgia coastal waters from shoreline out to the 200 m isobaths (majority of sightings between shoreline and 20 m isobath), respectively (Hayes et al., 2017). Previous research on the SGES Stock was primarily in the BSE waters from the Turtle and Brunswick Rivers northward to Altamaha Sound (Balmer et al., 2011) (Figure 2). For this study, dolphins were targeted for health assessment and tagging in the BSE waters south of the Turtle and Brunswick Rivers, and the adjacent coastal waters from Altamaha Sound south to the Georgia/Florida state border. All field efforts were based out of the Georgia Department of Natural Resources (GDNR) office in Brunswick, Georgia. Weather conditions heavily influenced daily capture locations. In coastal waters, health assessment operations were attempted in Beaufort Sea State (BSS) <3 and minimal swell. On field days in which conditions did not permit coastal captures, health assessments were carried out in BSE waters, initially targeting animals farther south and progressively working closer to the field base as the day progressed.

### 2.2 Dolphin handling

**Figure 3:**
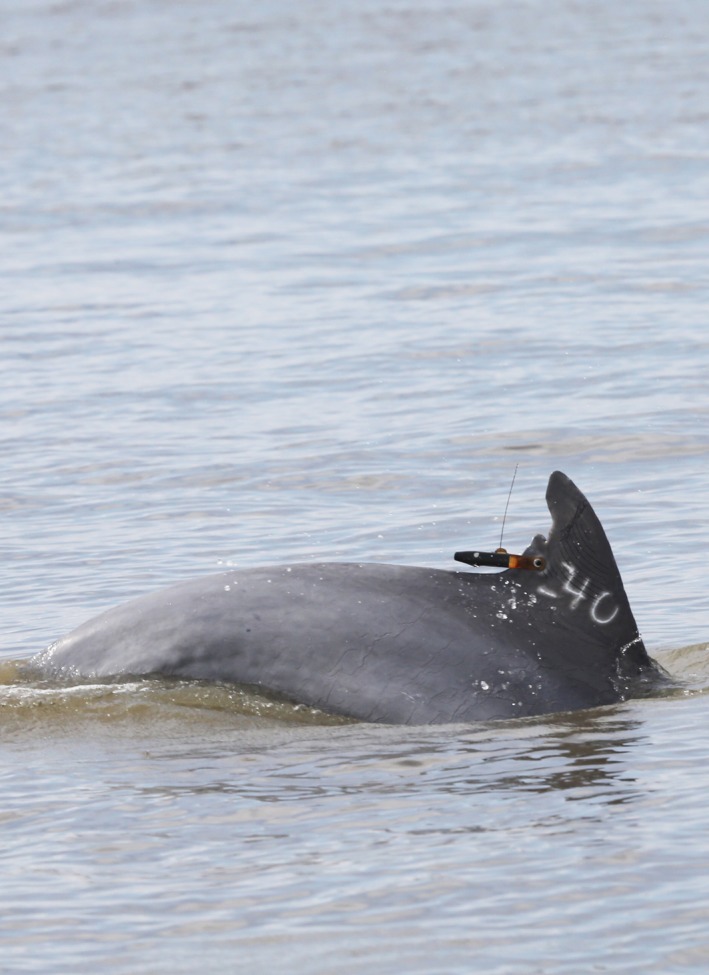
Male, 17 years old, common bottlenose dolphin, Z40, with SPOT‐299A satellite transmitter (Wildlife Computers, Redmond, WA, USA)

The 2015 dolphin health assessment was conducted over 10 field days between 14 and 25 September. Capture‐release methodologies have been detailed for previous studies (Asper, 1975; Norman, Hobbs, Foster, Schroeder, & Townsend, 2004). Briefly, one to two dolphins were encircled with a 366 m by 7 m deep seine net. For the majority of capture sets, water depth (>2 m) and bottom substrate type (heavy mud and oyster shell) prevented shallow‐water capture protocols. Instead, once an animal became entangled in the net, it was handled from one of three 6.3 m, center‐console, Zodiac (Zodiac Milpro International, Paris, France) rigid‐hulled inflatable boats (RhIBs) with twin 90‐hp four stroke outboard engines. Once restrained, the animal was moved onto a 3 m long, tri‐fold floating mat. Sex was determined for all dolphins and any female dolphin greater than or equal to 220 cm was held on the floating mat until an ultrasound exam could be conducted to determine if the dolphin was pregnant (Smith et al., 2013). Abbreviated sampling was conducted for any diagnosed pregnant females, or dolphins that were becoming overly stressed (i.e., rapid respirations, greater than 8 breaths per minute, or arching), or if weather deteriorated to unworkable conditions. Samples were either collected while the animal was on the floating mat or on a specially designed processing vessel (R/V *Megamouth*, a 9.1 m Munson “Packman” monohull; William E. Munson Company, Burlington, WA, USA). Prior to release, dolphins were freeze‐branded with a letter (Z) and two‐digit number (♀, odd; ♂, even) to provide long‐term identification (Scott, Wells, Irvine, & Mate, 1990) (Figure 3).

### 2.3 Sample collection, processing, and analyses

#### 2.3.1 Dolphin Morbillivirus (DMV)

Blood was collected from the ventral fluke vasculature using a 19 g × ¾” butterfly catheter. For serum samples, blood was spun in the field (E8 Fixed‐Speed Centrifuge, LW Scientific, Lawrenceville, GA, USA), approximately 30–45 min post‐collection to allow for clot to form. Blowhole and rectal swabs were collected using sterile polystyrene tipped swabs that were inserted into the dolphin's blowhole during a breath or into the dolphin's anus, respectively. Serum, blowhole and rectal samples for DMV testing were stored in a liquid nitrogen (N_2_) dry shipper at approximately −190°C in the field and in a −80°C freezer at the National Institute of Standards and Technology (NIST) Specimen Bank (Charleston, SC, USA). Serum was shipped to the Marine Mammal Diagnostic Service (Athens Veterinary Diagnostic Laboratory, University of Georgia, Athens, GA, USA) for DMV serology. Blowhole and rectal swabs were shipped to the University of California at Davis Marine Ecosystem Health Diagnostic and Surveillance Laboratory (Davis, CA, USA) for DMV polymerase chain‐reaction (PCR) testing. DMV titers were measured using the virus neutralization test (VNT) as previously described (Saliki & Lehenbauer, 2001). The Belfast strain of DMV was used. Twofold dilutions of serum (25 μL) were made in 96‐well microtiter plates with Dulbecco's minimum essential medium (DMEM). An equal volume of virus (25 μL) containing approximately 100 TCID50 was added to each well and plates were incubated at 35.5°C for 1 hr. Vero Dog Slam cells (1.5 × 104 cells in 150 μL) were added to each well and the plates incubated at 35.5°C in 5% CO_2_ for 3 days, after which they were examined for cytopathic effects. The antibody titer was defined as the highest dilution of serum that neutralized CPE. Results were expressed as positive if titers were ≥1:16 (Rowles et al., 2011). Frozen blowhole and rectal swabs were tested for cetacean morbillivirus by reverse transcriptase‐PCR (RT‐PCR). RNA was extracted using TRIzol reagent (Invitrogen Corp., Carlsbad, CA, USA), and cDNA was transcribed using the Superscript III First Strand kit (Invitrogen Corp.). DMV testing was performed using universal morbillivirus primers targeting a 429 base pair fragment of the phosphoprotein (P) gene (Barrett et al., 1993) followed by nested primers specific for dolphin morbillivirus. Bands of correct size were excised and purified PCR products were cloned (pCR4‐TOPO vector; Invitrogen Corp.) and sequenced (ABI 3,730 Capillary Electrophoresis Genetic Analyzer; Applied Biosystems, Inc., Foster City, CA, USA). Partial sequences of the P gene were compared against morbilliviral sequences available in the GenBank database. A tooth was extracted for age determination (Hohn, Scott, Wells, Sweeney, & Irvine, 1989; McFee, Adams, Fair, & Bossart, 2012; Ridgeway, Green, & Sweeney, 1975) as conditions permitted from dolphins that were not pregnant or identified by the veterinary team to be overly stressed.

#### 2.3.2 Satellite telemetry

Prior to release, all dolphins were tagged with either a SPOT‐299A (location) or SPLASH10–268D (location‐time‐depth) satellite transmitter (Wildlife Computers, Redmond, WA, USA) (Figure 3). Satellite transmitter specifications, programing, and attachment protocols have been reviewed in detail in previous studies (Balmer, Wells, Howle, et al., 2014a; Wells et al., 2017). Briefly, the SPOT‐299A and SPLASH10‐268D tags had a projected battery life of 280 days and 130 days (250 transmissions per day), respectively. To increase battery life and provide the highest quality location data, transmitters were programmed for seven, 1‐hr transmission windows per day in the Argos Collecte Localisation Satellites (CLS) system (Collecte Localisation Satellites, 2011), specifically targeting transmission windows with optimal satellite pass durations and daylight hours to permit a field team to relocate tagged animals as necessary.

SPLASH dive depth and dive duration data were transmitted in the bandwidth‐conserving format of Time‐at‐Depth (TAD) and dive duration histograms, in which the percentage of time spent within user‐defined categories was determined. TAD categories were grouped into the following bins: <2, 2–3, 3–4, 4–5, 5–6, 6–7, 7–8, 8–9, 9–10, 10–15, 15–20, 20–50, >50 m. Dive duration categories were grouped into the following bins: <30, 30–60, 60–90, 90–120, 120–150, 150–180, and >180 s.

Tag attachment distance was 38.4 mm from the trailing edge of the dorsal fin, and tags were affixed in the lower third of the dorsal fin. Tags were coated with Propspeed (Oceanmax, Ltd., Auckland, NZ) (excluding the saltwater switches) to reduce biogrowth. Vessel‐based, photo‐ID surveys were conducted several times across the duration of the tags to locate tagged individuals, assess dolphin and tag condition, and identify reasons for tag failure. In addition, photographs were received from the public and used for assessment of dolphin and tag condition. Mode of tag failure (e.g., attachment, battery, or biogrowth) was determined based upon parameters defined in Balmer, Wells, Howle, et al. (2014a).

All telemetry data were received from the Argos CLS system and filtered through the Douglas Argos‐filter algorithm (Douglas, 2006). Location class (LC) 3 and 2 data were used for subsequent spatial analyses, with estimated errors of <250 m and 250–500 m, respectively. All telemetry data were plotted in ArcMap 10.4.1 (ESRI, Redlands, CA, USA). Strahler Stream Order (SSO) was used to classify all locations by tributary size (Strahler, 1952). Tributaries measured with SSO encompassed small creeks (first order), large creeks (second order), rivers (third order), sounds (fourth order), and ocean (fifth order) (e.g., Balmer et al., 2013). Each dolphin was classified into one of three ranging patterns based upon their telemetry locations within SSO tributaries: Estuary‐ SSO 1–3; Sound‐ SSO 2–5; Coastal‐ SSO 3–5.

Individual and cumulative ranging patterns were defined as 95% and 50% utilization distributions (UDs), which are probability distributions of an animal’s or group of animals’ movements (use) in the available habitat (plane) (Worton, 1989). UDs summarize ranging pattern data and provide insight into habitat use, spatiotemporal overlap, and short‐term site fidelity (reviewed in Fieberg & Kochanny, 2005). Kernel density estimates (KDEs) are a quantitative method to determine UDs (Worton, 1989). For individual and cumulative UDs, a KDE method for an environment with barriers to movement in Geostatistical Analyst and Spatial Analyst Tools (ArcGIS 10.4.1) was used. All spatial analyses were calculated in the Universal Transverse Mercator (UTM) Zone 17 North projection and the World Geodetic System (WGS) 1984 datum. The output grid cell size was 250 × 250 m to account for Argos estimated LC 3 and 2 errors and to allow for fine‐scale spatial resolution of the telemetry data (e.g., Jay, Fischbach, & Kochnev, 2012, Sprogis, Raudino, Rankin, MacLeod, & Bejder, 2016).

The selection of bandwidth, or the smoothing parameter (*h)*, is an important decision in which KDE distributions can be over‐ or under‐estimated depending on this value (Horne & Garton, 2006; Kie et al., 2010). The methodology for bandwidth selection is dependent on the goals of the project, ranging patterns of the target species, and amount of data available for spatial analyses (Gitzen, Millspaugh, & Kernohan, 2006; Rayment et al., 2009). A rule‐based ad hoc method (Kie, 2013) and Home Range Tools (HRT) for ArcGIS (Rodgers, Kie, Wright, Beyer, & Carr, 2015) were used to determine the appropriate bandwidth for KDEs of each individual and cumulative ranging pattern. UDs (95% and 50%) were then determined from these KDEs. Spatial overlap among ranging patterns was calculated following methods described in MacLeod (2013).

## 3 RESULTS

### 3.1 Dolphin health assessment, dolphin morbillivirus (DMV) prevalence and satellite tagging

During September 2015, 19 dolphins (♀ = 4, ♂ = 15) were captured, assessed, and tagged with satellite transmitters (SPOT‐299A, *N* = 14; SPLASH10–268D, *N* = 5) in coastal (*N* = 9) and estuarine (*N* = 10) waters of southeastern Georgia (Table 1; Figure 2). Five of these dolphins were previously sampled via remote biopsy (Z34, Aug. 2004; Z40, Mar. 2007; Z42; Aug. 2006, Mar. 2007; Z44, Mar. 2007; and Z48, Mar. 2007) within several kilometers of their capture locations (P. Rosel personal communication). These five remote biopsied dolphins and two other individuals (Z30 and Z50) have been sighted across multiple seasons and years based upon long‐term (2004–2016) photo‐ID effort in the region (Balmer et al., 2011). Age was determined for eight of the 15 male dolphins through tooth longitudinal‐sectioning and ranged from 5 to 30 years old. Of the four female dolphins handled, one (Z37) was pregnant (1st trimester). Four of the 19 dolphins tested positive for DMV antibodies in serum (Table 1) and titers ranged from 1:32 to 1:256. All dolphins were negative for DMV by PCR in blowhole and rectal swabs.

**Table 1:** Capture‐telemetry summary for common bottlenose dolphins tagged in the coastal and estuarine waters of Georgia during September 2015, grouped by ranging pattern classification, and including dolphin morbillivirus (DMV) prevalence and (50% and 95%) utilization distributions (UDs)

FB	Sex	Age	Length (cm)	Weight (kg)	Mbv Positive (≥1:16)	# of locations (LC 3 and 2)	Number of Days Transmitting	50% UD (km^2^)	95% UD (km^2^)	Reason for Tag Failure
Coastal ranging pattern classification										
Z28	M	ND	211	ND	Yes	182	129	180	1,135	Attachment
Z44	M	26	265	220	No	194	94	81	447	Unknown
Z46	M	12	225	ND	Yes	83	123	276	1,227	Antenna
Sound ranging pattern classification										
Z30	M	ND	228	126.8	No	271	144	44	210	Unknown
Z33	F	ND	214	ND	No	364	181	25	148	Unknown
Z34	M	ND	250	176.2	No	263	72	40	311	Attachment
Z35	F	ND	256	ND	No	156	98	10	73	Unknown
Z40	M	17	242	170	No	163	160	51	210	Unknown
Z42	M	15	217	160	No	318	90	40	208	Attachment
Z48	M	ND	251	210	Yes	181	108	38	377	Attachment
Z50	M	30	273	233	Yes	237	127	11	84	Attachment
Z54	M	ND	255	ND	No	236	125	57	138	Biogrowth
Z56	M	ND	226	ND	No	138	52	13	64	Unknown
Estuary ranging pattern classification										
Z31	F	ND	206	87.2	No	152	105	8	22	Unknown
Z32	M	ND	210	ND	No	244	145	8	39	Attachment
Z36	M	18	255	177	No	467	172	5	43	Unknown
Z37	F	ND	251	162.5	No	337	148	11	33	Antenna
Z38	M	7	206	98.4	No	172	128	5	37	Unknown
Z52	M	5	202	93.2	No	159	169	2	30	Unknown

### 3.2 Satellite telemetry

**Figure 4:**
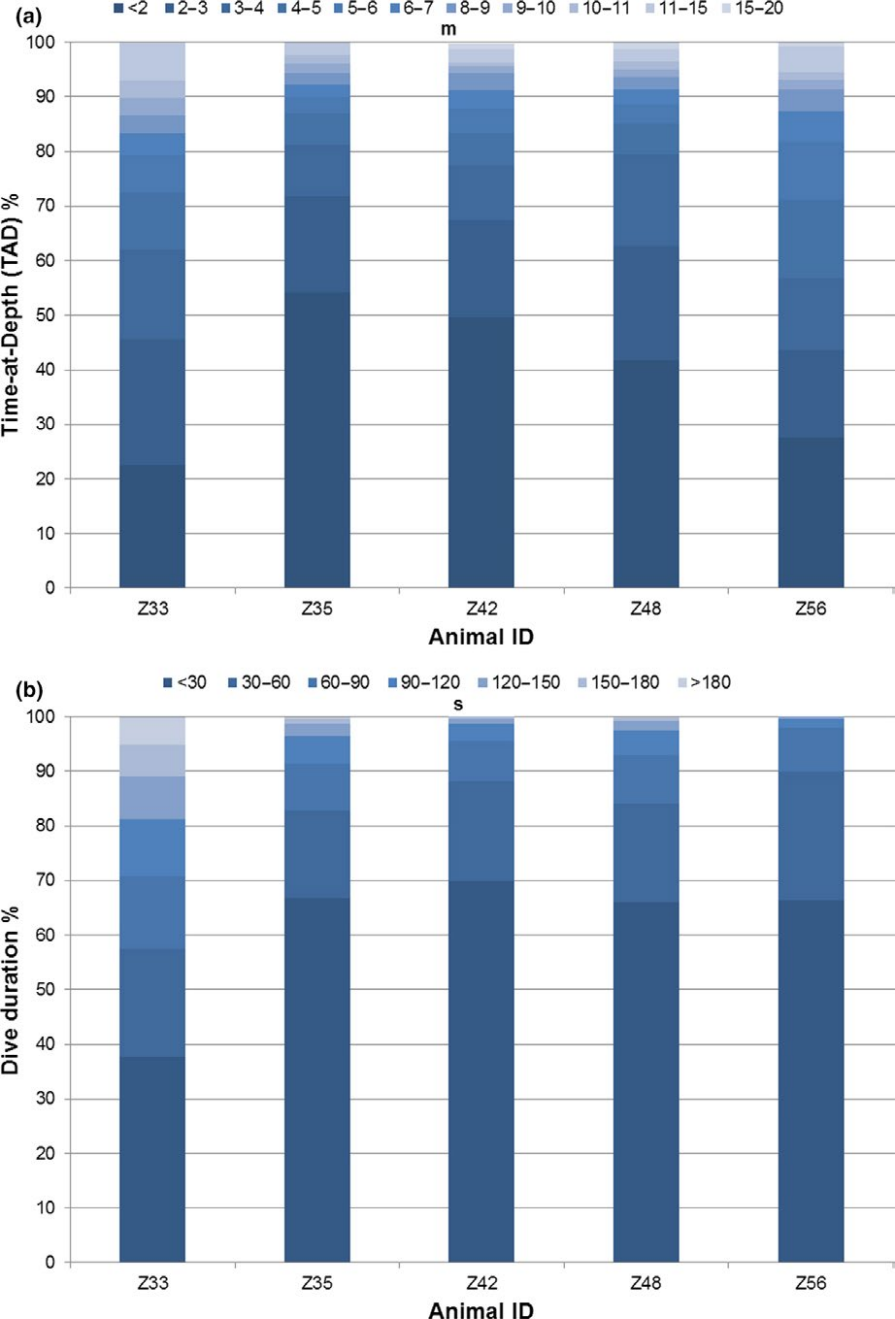
Time‐at‐Depth (TAD) (a) and dive duration (b) percentages for the five common bottlenose dolphins tagged with SPLASH transmitters

SPOT‐299A, location‐only tags (♀ = 2, ♂ = 13) and SPLASH10–268D, location‐time‐depth tags (♀ = 2, ♂ = 3) transmitted for a mean of 133 ± 28 *SD* days and 101 ± 43 *SD* days, respectively. The mean number of locations (LC 3 and 2) per tag duration and on an individual day was 125 ± 35 *SD* and 1.91 ± 0.79, respectively. Mode of tag failure was identified for 9 tags (attachment, *N* = 6; antenna, *N* = 2; and biogrowth, *N* = 1). For the five dolphins tagged with SPLASH transmitters, TAD ranged from <2 to 15–20 m bins with the majority of dives in the <2 and 2–3 m bins (Figure 4a). Dive duration ranged from <30 to >180 s bins with the majority of dives <30 s and 30–60 s (Figure 4b). TAD and dive duration data were generally comparable across all five tagged dolphins.

**Figure 5:**
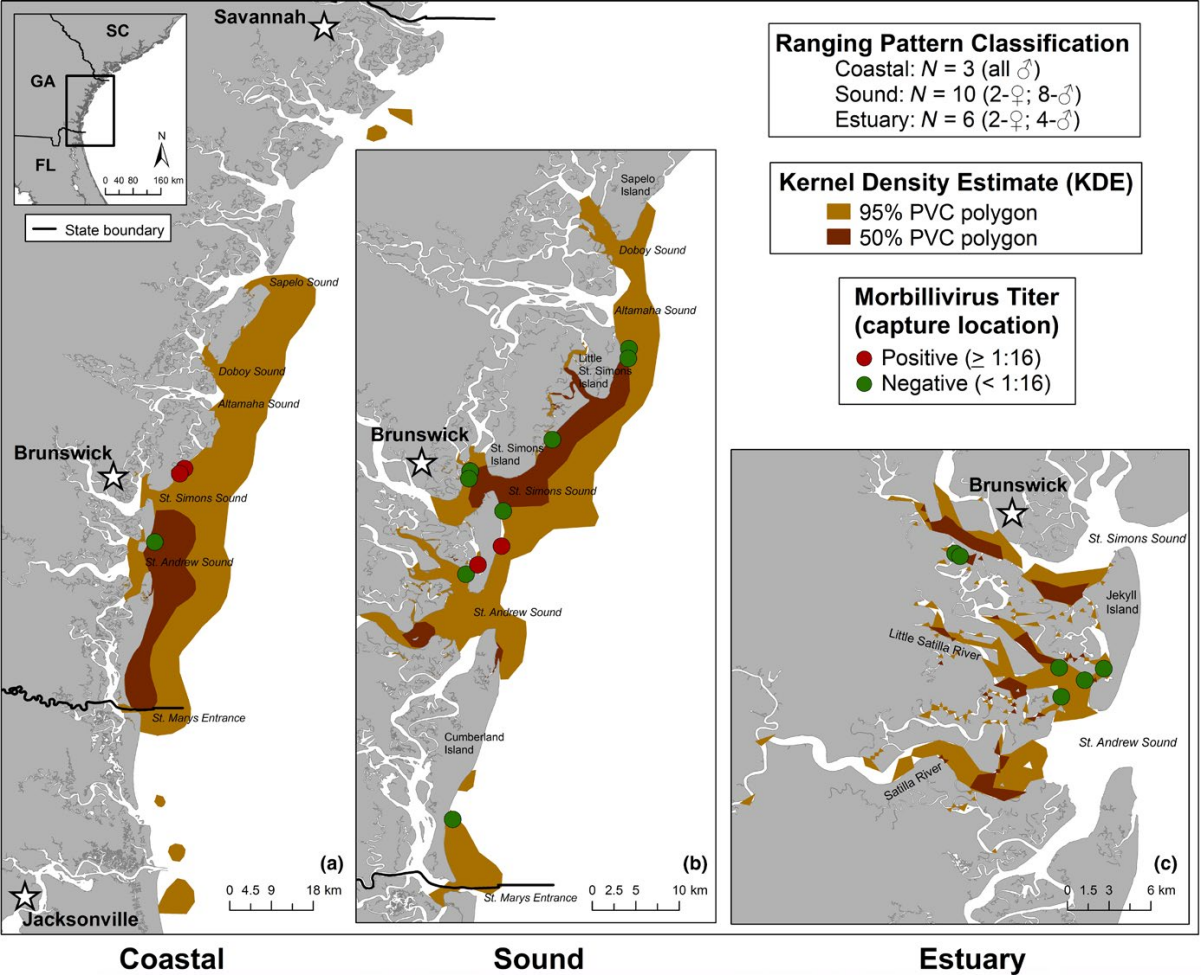
50% and 95% utilization distributions (UDs) and capture location with dolphin morbillivirus (DMV) titer for the Coastal, Sound, and Estuary ranging patterns

Individual UDs ranged from 2 to 276 km^2^ (50%) and 22–1,227 km^2^ (95%) and extended as far as 32 km up the Satilla River and 15 km offshore of St. Simons and St. Andrew Sounds (Table 1; Figure 5). For cumulative ranging pattern UDs, data from September 2015 through January 2016 were included in which the majority of tags transmitted through this duration. The Coastal ranging pattern had the largest UDs, followed by Sound and Estuary ranging patterns (Table 2; Figure 5).

**Table 2:** 50% and 95% utilization distributions (UDs) and percentage of overlap area (95% UD) for Coastal, Sound, and Estuary ranging patterns

				Overlap Area; 95% UD (%)		
Ranging pattern	*N*	50% UD (km^2^)	95% UD (km^2^)	CST	SND	EST
Coastal	3	242	1,231		24	1
Sound	10	75	371	80		11
Estuary	6	16	65	12	63	

**Figure 6:**
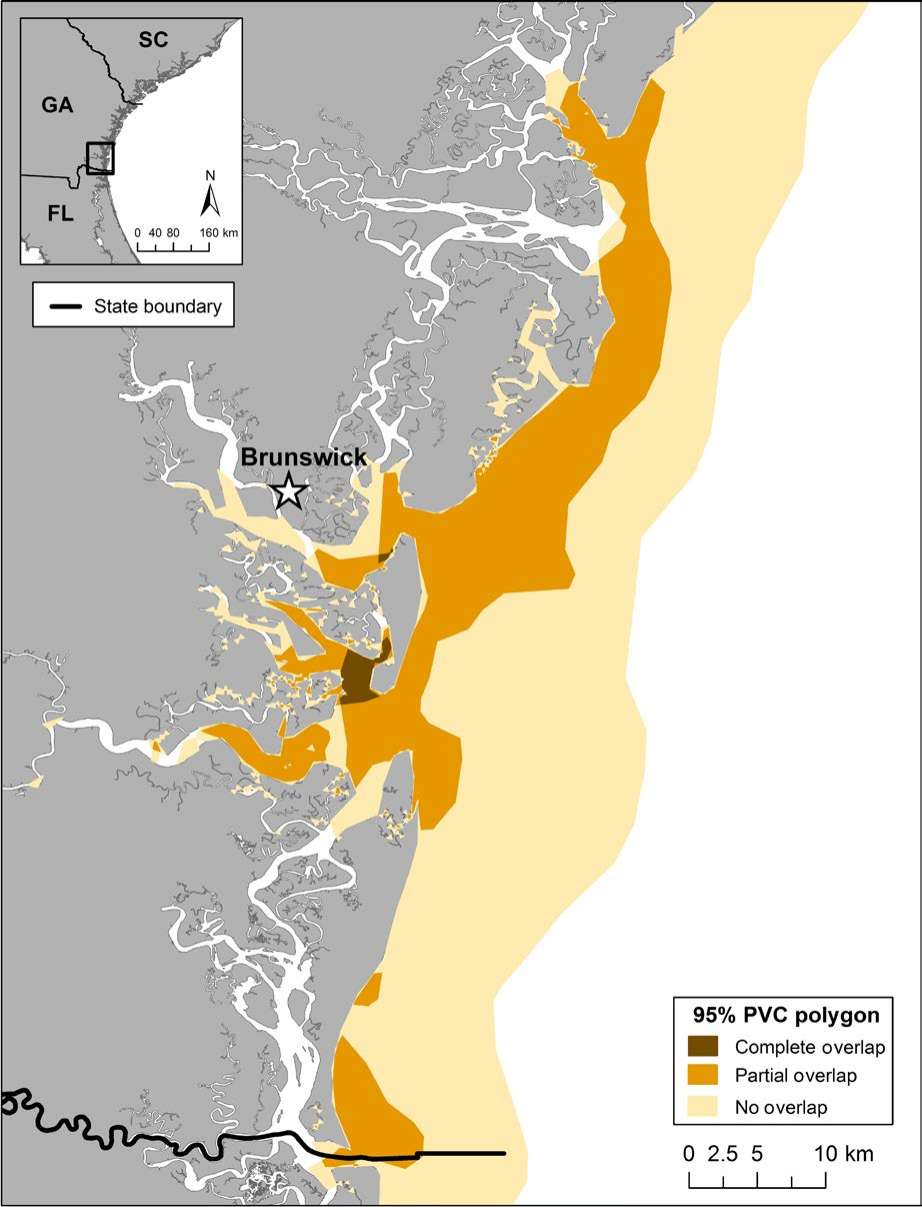
Spatial overlap across 95% utilization distributions (UDs) for the Coastal, Sound, and Estuary ranging patterns; Complete Overlap—all three ranging patterns, Partial Overlap—two of the three ranging patterns, No Overlap—one of the three ranging patterns

Coastal dolphins had the highest prevalence of antibodies to DMV (0.67; *N* = 2/3), followed by Sound (0.20; *N* = 2/10) and Estuary (0.00; *N* = 0/6) dolphins (Table 1; Figure 5). All ranging patterns had some degree of 95% UD overlap with each other (Table 2; Figure 6). The Coastal ranging pattern overlapped 24% with the Sound and 1% with the Estuary ranging patterns. The Sound ranging pattern overlapped 80% with the Coastal and 11% with the Estuary ranging patterns. The Estuary ranging pattern overlapped 12% with the Coastal and 63% with the Sound ranging patterns. There was complete overlap of all three ranging patterns in two small areas within St. Simons and St. Andrew Sounds (Figure 6).

## 4 DISCUSSION

During the 2013–2015 DMV outbreak, over 1,600 dolphins stranded, of which a minimum of 186 of the 207 dolphins tested were confirmed positive or suspected positive for DMV (Fauquier, Goldstein, Colegrove, Rotstein, & DiGiovanni, 2014). Classification of individual stranded dolphins to their respective BSE or coastal stock, spatial overlap between stocks, and prevalence of exposure to DMV within stocks prior to this mortality event were for the most part unknown. Ranging patterns and DMV antibody prevalence were determined for 19 free‐ranging common bottlenose dolphins in this study. Although caution should be used in interpreting the results with this limited number of individual dolphins, numerous studies have used comparable sample sizes to assess health and ranging patterns of large, marine vertebrates (e.g., Elwen et al., 2006, Meyer, Clark, Papastamatiou, Whitney, & Holland, 2009, Schwacke et al., 2012, Lane et al., 2015). This study provided some of the first insights into ranging patterns for dolphins in the BSE and coastal waters of Georgia, estimated DMV antibody prevalence following the 2013–2015 DMV outbreak, and quantified spatial overlap to assess differences between ranging patterns to assess animal movement and contact DMV transmission.

### 4.1 Stock structure

Stocks of marine mammals have been primarily delimited using genetic analyses (Rosel, Forgetta, & Dewar, 2005), but, additional sampling techniques such as photo‐ID and telemetry have been used to test and assist in classification of individuals into their respective stocks (Balmer, Wells, Schwacke, et al., 2014b; Sveegaard et al., 2015). In the present study, both Estuary and Sound dolphins had small to moderate UDs (Tables 1 and 2; Figure 5b,c) and many individuals had long‐term photo‐ID sighting histories (2004–2016), suggesting high site fidelity to localized BSEs and adjacent coastal waters. These individuals are likely long‐term residents but could represent two sub‐populations of the SGES stock with different habitat preferences (e.g., Lusseau et al., 2006, Wiszniewski, Allen, & Möller, 2009). In the southeastern U.S., similar sub‐populations have been identified for dolphins in more interior estuarine waters and those in larger sounds and surrounding barrier islands (Urian, Hofmann, Wells, & Read, 2009; Wells et al., 2017). Estuary dolphins had UDs (34 ± 8 km^2^; mean ± *SD*; Table 1) generally comparable to dolphin UDs determined via photo‐ID and telemetry in other BSE stocks in the southeastern U.S. (McHugh, Allen, Barleycorn, & Wells, 2011; Owen, Wells, & Hofmann, 2002; Urian et al., 2009; Wells et al., 2017). Conversely, Sound dolphins had UDs (182 ± 103 km^2^; mean ± SD; Table 1) several times larger than Estuary and other southeastern U.S. BSE UDs, and included coastal waters primarily within 5 km from shore (approximately 75% of satellite locations), but did have limited ranges extending over 10 km offshore.

Coastal dolphins were characterized by large UDs that included the coastal waters of Savannah, Georgia to Jacksonville, Florida primarily within 10 km from shore (approximately 75% of satellite locations), but did have limited ranges extending to over 15 km offshore (Tables 1 and 2; Figure 5a). None of the three Coastal dolphins were previously identified in the long‐term photo‐ID catalog for this region. However, historical photo‐ID effort was primarily in BSE waters. The 2015 health assessment was conducted in September when the Southern Migratory Coastal Stock was hypothesized to be farther north off North Carolina (Hayes et al., 2017; Silva, 2016; Urian, 2016). Based upon Coastal dolphin movements and the capture/tagging timeframe, these individuals are likely members of the SC‐GA Coastal Stock. The large UDs of Coastal dolphins (936 ± 426 km^2^; mean ± *SD*; Table 1) create a logistical challenge for a comprehensive assessment of the SC‐GA Coastal Stock's ranging pattern. In addition to health assessment/satellite tagging locations targeting coastal waters, photo‐ID comparisons between projects/field sites (e.g., Balmer et al., 2016, Urian, 2016) and survey effort including both coastal and BSE waters (e.g., Laska, Speakman, & Fair, 2011, Silva, 2016) can provide data essential in determining ranging patterns, site fidelity, and stock discreteness of the SC‐GA Coastal Stock.

### 4.2 Dolphin morbillivirus (DMV) and spatial overlap

Determining spatial overlap of exposed and naive hosts is essential for predicting the spread of infectious diseases (Robert et al., 2012). Preliminary modeling efforts for the recent western North Atlantic DMV outbreak concluded that information on movements and interactions among different stocks was one of the greatest needs for refining the model in order to better understand the dynamics of the outbreak (Morris et al., 2015). Here, we provide the first data on movement, spatial overlap, and DMV antibody titers for surviving dolphins following that outbreak.

Rowles et al. (2011) previously documented that DMV antibodies decrease over time, but this trend may not always be consistently observed across all individuals. In this study, three of the sampled dolphins had relatively high DMV titers (1:256) and at least two of these were too young to have been exposed during the previous 1987–1988 outbreak; Z46 was only 12 years old, and Z28, with a length of only 211 cm, was likely less than 10 years old (McFee, Schwacke, Stolen, & Mullin, 2010). It is therefore likely that these dolphins were exposed during the more recent outbreak, although the potential for exposure outside of an outbreak is also possible. The fourth DMV positive has a much lower titer (1:32), and at 30 years old, could have potentially been exposed during the prior 1987–1988 outbreak.

Prevalence of DMV antibodies differed for the Estuary and Sound dolphins, which are putatively part of the SGES Stock, versus the Coastal dolphins, which are putatively the SC‐GA Coastal Stock. Combining the BSE and Sound ranging groups, the prevalence of positive titers in SGES dolphins was 0.13 (*N* = 2/16), which was significantly lower than the prevalence of 0.67 (*N* = 2/3) in SC‐GA Coastal dolphins, suggesting that contact rates may vary between BSE and coastal stocks. Although the sample size for these hypothesized SGES and SC‐GA Coastal dolphins is low, these results are supported by prior studies of the nearby (<200 km northeast) Charleston Estuarine System Stock, which reported a zero prevalence of DMV and Porpoise Morbillivirus (PMV) antibodies for dolphins sampled in 1999 (*N* = 0/14, 0.00–0.20 95% CI; Rowles et al., 2011) and for dolphins sampled in 2003–2005 (*N* = 0/83; Bossart et al., 2010). In contrast, Duignan et al. (1996) identified a high prevalence of DMV antibodies (0.88, 0.77–0.99 95% CI) in dolphins sampled off Virginia (hypothesized to be members of the Northern Migratory Coastal Stock) during the 1987–1988 DMV outbreak. Although DMV antibody prevalence and ranging patterns for the Southern Migratory Coastal Stock are not well understood, it is hypothesized that in addition to this stock overlapping seasonally with the Northern Migratory Coastal Stock, it also overlaps with the SC‐GA Coastal Stock during January–March as it migrates along the western North Atlantic coast (Hayes et al., 2017). In this study, Coastal dolphins not only had the highest prevalence of DMV antibodies (0.67; *N* = 2/3), but also the largest UDs (approximately 200 km of coastline) (Tables 1 and 2; Figure 5a). These results suggest that contact rates between coastal stocks could be relatively high, at least seasonally, with members of the SC‐GA Coastal Stock exposed to DMV as the Southern Migratory Coastal Stock migrates through the region.

Within the SGES Stock, the prevalence of positive DMV titers was higher for dolphins of the Sound ranging pattern (0.20; *N* = 2/10) as compared to that of the Estuary ranging pattern and Estuary (0.00; *N* = 0/6), although once stratified by ranging pattern, sample sizes were small. Differences in spatial overlap may provide insight into the gradient of DMV exposure identified across ranging patterns. The majority of the Sound dolphins’ range (80%) overlapped with Coastal dolphins, suggesting higher contact rates between infected Coastal dolphins (Southern Migratory and/or SC‐GA Coastal Stocks) and Sound dolphins. In contrast, only 12% of the Estuary dolphins’ range overlapped with Coastal dolphins, suggesting low contact rates between coastal stocks and Estuary dolphins. The moderate spatial overlap (63%) between Estuary and Sound dolphins may be a source of exposure for future disease outbreaks that begin in coastal waters and then impact different sub‐populations within the SGES Stock or potential other BSE Stocks.

Although numerous studies across a variety of taxa have linked spatial overlap and disease transmission rates (reviewed in Robert et al., 2012), social barriers (i.e., group size and interaction rates) (Loehle, 1995) may be an additional factor influencing DMV prevalence. In the western North Atlantic, dolphin group size is higher in coastal waters than within BSEs (Speakman et al., 2006; Torres, Mclellan, Meagher, & Pabst, 2005; Toth et al., 2011) and thus contact rates are likely greater between members of stocks that are found along the coast. Laska et al. (2011) used photo‐ID data to identify high interaction rates (69% of cataloged individuals observed in mixed groups) for dolphins in coastal waters off Charleston, South Carolina, which were likely members of the SC‐GA Coastal Stock, as compared to dolphins in the adjacent BSE stock (Charleston Estuarine System). The health assessment and telemetry data along the Georgia coast are consistent with the photo‐ID data from Charleston, suggesting that spatial overlap and interactions between dolphin sub‐populations and/or stocks could influence disease prevalence among the SC‐GA Coastal and parapatric BSE Stocks. However, little is known about spatial overlap and social barriers between the Southern Migratory Coastal Stock and other coastal as well as BSE stocks. Qualitative observations suggest that both the Northern and Southern Migratory Coastal Stocks are sighted farther offshore, surface in synchronous, large, tight groups, and travel along the coast at a faster rate than other coastal or BSE stocks (Zolman pers. comm., Barco, Swingle, McLellan, Harris, & Pabst, 1999). DMV is likely spread via physical contact or inhalation (Black, 1991; Van Bressem et al., 1999), thus these characteristics may increase contact rates within migratory coastal stocks while conversely lessening disease transmission to other stocks. Future research investigating spatial overlap and behavior of migratory stocks is essential to better understand disease transmission amongst western North Atlantic dolphin stocks. Conventional encirclement methods of capturing dolphins for health assessment and satellite tag attachment are logistically challenging in coastal waters where the Northern and Southern Migratory Coastal Stocks are located. Developing alternative capture approaches (e.g., hoop‐netting; Klatsky, Wells, & Sweeney, 2007) and novel methods to remotely tag individuals from these stocks will provide data necessary for a comprehensive assessment of contact rates and disease prevalence.

## 5 CONCLUSIONS

This study provides the first data from dolphins that were exposed to and survived (as evidence from presence of DMV antibodies) the 2013–2015 DMV outbreak, as well as telemetry data to compare spatial overlap of a BSE and a coastal stock within the region of the outbreak. Our results support previous findings from a modeling effort of broader disease transmission during the outbreak, which used data from stranded dolphins (Morris et al., 2015), and suggested local movements may dominate spatial spread, with broader dissemination driven by seasonal migratory movements of coastal stocks. Consistent with the Morris et al. (2015) model, we found that along the Georgia coast, probability of exposure may be influenced by fine‐scale spatial use, and that dolphins in the SC‐GA Coastal Stock, which are more likely to interact with the Southern Migratory Coastal Stock, have a higher prevalence of DMV antibodies indicating previous exposure to the virus. The SC‐GA Coastal Stock may be more susceptible to DMV spread via the Southern Migratory Coastal Stock along the coast. However, the SGES should be considered a stock of concern with a small population estimate (*N* = 194; CV = 0.05) (Hayes et al., 2017), and extremely high PCB levels (Balmer et al., 2011; Kucklick et al., 2011), which have been documented to adversely affect the immune system and may facilitate the emergence of infectious disease (e.g., Ross, 2002). In fact, previous health assessments of the SGES stock documented a decrease in immune function associated with increasing PCB levels (Schwacke et al., 2012). Therefore, while exposure risk from a future outbreak may be lower for the SGES stock, the fact that the majority of the stock are naïve to DMV, and may additionally exhibit suppressed immune response, suggests that this population may be particularly vulnerable. Future health assessment and telemetry projects in the coastal waters are essential to better assess contact rates and disease prevalence among western North Atlantic coastal and BSE stocks.

### 5.1 NOAA disclaimer

This publication does not constitute an endorsement of any commercial product or intend to be an opinion beyond scientific or other results obtained by the National Oceanic and Atmospheric Administration (NOAA). No reference shall be made to NOAA, or this publication furnished by NOAA, to any advertising or sales promotion which would indicate or imply that NOAA recommends or endorses any proprietary product mentioned herein, or which has as its purpose an interest to cause the advertised product to be used or purchased because of this publication.

## CONFLICT OF INTEREST

None declared.

## AUTHOR CONTRIBUTIONS

Dolphin health assessments require a great deal of funding, logistical support, and a multi‐faceted team to link all of the data into a comprehensive manuscript. The authors are a mix of field and laboratory researchers that at a minimum did two of the following: 1) preparations and collection of the field data [Brian Balmer (BB), Eric Zolman, (EZ), Teri Rowles (TR), Cynthia Smith (CS), Forrest Townsend (FT), Deb Fauquier (DF), Clay George (CG), Larry Hansen (LJ), Brian Quigley (BQ), Wayne McFee (WM), Jeanine Morey (JM), Todd Speakman (TS), and Lori Schwacke (LS)], 2) laboratory analyses [Tracey Goldstein (TG), WM, Patricia Rosel (PR), and Jerry Saliki (JS)], 3) spatial analyses (BB), 4) synthesis of data (BB, EZ, TR, CS, FT, DF, LH, BQ, JM, PR, TS, and LS), and 5) drafted sections of the manuscript (BB, DF, TG, JS, and LS).

## DATA ACCESSIBILITY

All satellite telemetry, time depth, and DMV data are located on ResearchGate (https://doi.org/10.13140/RG.2.2.35575.47523).
